# vB_PaeM_MIJ3, a Novel Jumbo Phage Infecting *Pseudomonas aeruginosa*, Possesses Unusual Genomic Features

**DOI:** 10.3389/fmicb.2019.02772

**Published:** 2019-11-28

**Authors:** Mohammed Imam, Bandar Alrashid, Faizal Patel, Ahmed S. A. Dowah, Nathan Brown, Andrew Millard, Martha R. J. Clokie, Edouard E. Galyov

**Affiliations:** ^1^Department of Respiratory Sciences, College of Life Sciences, University of Leicester, Leicester, United Kingdom; ^2^Laboratory Department, University Medical Center, Umm Al-Qura University, Mecca, Saudi Arabia; ^3^Department of Genetics and Genome Biology, College of Life Sciences, University of Leicester, Leicester, United Kingdom; ^4^King Faisal Specialist Hospital and Research Centre, Riyadh, Saudi Arabia

**Keywords:** bacteriophages, *Pseudomonas aeruginosa*, jumbo phages, intein, host range analysis, antibiotic resistance, phylogenetic analysis

## Abstract

Phages are the most abundant biological entity on Earth. There are many variants in phage virion sizes, morphology, and genome sizes. Large virion sized phages, with genome sizes greater than 200 kbp have been identified and termed as Jumbo phages. These phages exhibit certain characteristics that have not been reported in phages with smaller genomes. In this work, a jumbo phage named MIJ3 (vB_PaeM_MIJ3) that infects *Pseudomonas aeruginosa* PAO1 was isolated from an equine livery yard in Leicestershire, United Kingdom. The genome and biological characteristics of this phage have been investigated. MIJ3 is a Myovirus with multiple long tail fibers. Assessment of the host range of MIJ3 revealed that it has the ability to infect many clinical isolates of *P. aeruginosa.* Bioinformatics analysis of the phage genome indicated that MIJ3 is closely related to the *Pseudomonas* phage, PA5oct. MIJ3 possesses several unusual features that are either rarely present in other phages or have not yet been reported. In particular, MIJ3 encodes a FtsH-like protein, and a putative lysidine synthase, TilS. These two proteins have not been reported in phages. MIJ3 also possesses a split DNA polymerase B with a novel intein. Of particular interest, unlike other jumbo phages infecting *Pseudomonas* spp., MIJ3 lacks the genetic elements required for the formation of the phage nucleus, which was believed to be conserved across jumbo *Pseudomonas* phages.

## Introduction

*Pseudomonas aeruginosa* is a widespread Gram-negative bacterium. It is an opportunistic pathogen and is the major cause of nosocomial infection with significant mortality rates. *P. aeruginosa* causes a wide variety of infections, ranging from self-limiting skin infections to life-threatening pneumonia and septicemia. Between January 2018 and January 2019, a total of 4,543 septicemia cases caused by *P. aeruginosa* were reported in England alone (Public Health England, 2019). The severity of *P. aeruginosa* infections is particularly high and problematic in immunocompromised patients with conditions such as cystic fibrosis (CF) and chronic obstructive pulmonary disease (COPD) (Gonçalves-de-Albuquerque et al., 2016). In fact, chronic pulmonary infections with *P. aeruginosa* is a major cause of death in CF patients (Ashish et al., 2012). With increasing rates of multiple-drug resistance (MDR) *P. aeruginosa* infections, phages are suggested as an alternative treatment solution, or to be combined with antibiotic, to treat bacterial infections (Krylov et al., 2015).

Bacterial viruses, bacteriophages (or phages), are the most abundant microorganisms in the environment (Suttle, 2005). Strikingly, it is estimated that almost half of the bacterial cells present in the environment are killed by phages every day (Potera, 2013). Phages are generally found in abundance where their hosts are found, and as such they are commonly found and isolated from environments such as freshwater, seawater, and animal manure (Clokie et al., 2011; Chaturongakul and Ounjai, 2015). The most common habitats for *P. aeruginosa* are water, soil, plants, and animals (Chatterjee et al., 2017). These sources are natural environments from which *Pseudomonas-*specific phages can be isolated, as phages coexist with their host cells (Shaburova et al., 2006; Krylov, 2014). There is strong evidence supporting the effectiveness of phage therapy in the treatment of lung infections associated with *P. aeruginosa* (Waters et al., 2017). Recent work by Cafora et al. (2019) shows the effectivity of phages against *P. aeruginosa* infection in a model of zebrafish with CF.

Phages vary in their size, morphology, and genomes. Examples of common and well-studied phages are the T4 Myovirus, which has a genome size of 168,903 bp, the T7 Podovirus which has a genome size of 39,937 bp, and the T5 Siphovirus which has a genome size of 121,752 bp. Phages with larger genome sizes are grouped as either jumbo or mega phages.

Phages with genome sizes over 500 kb are grouped collectively as megaphages (Devoto et al., 2019). To date, only 15 megaphages have been found, all of which infect bacteria from the *Prevotella* genus (Devoto et al., 2019). All of the megaphages were identified genomically from the analysis of viral metagenomics data; these phages have not been physically isolated yet. On the other hand, phages with genome sizes >200 kb, but <500 kb, have been termed jumbo phages (Yuan and Gao, 2017). Over 150 jumbo phage genomes have been deposited into the EBI and NCBI databases. The majority of them infect different Gram-negative bacteria, of which includes species from *Escherichia*, *Aeromonas*, *Caulobacter*, *Erwinia*, *Pseudomonas*, *Vibrio*, and *Salmonella*. Only six jumbo phages infecting Gram-positive bacteria have been characterized; all of which infect *Bacillus* species. A full list of megaphages and jumbo phages described to date, can be found in Supplementary Table 1.

At the time of this manuscript’s submission, the largest jumbo phages described are: *Bacillus* virus G with a genome size of 497,513 bp (Donelli et al., 1975), *Agrobacterium* phage Atu_ph07 with a genome size of 490,380 bp (Attai et al., 2018), and *Salicola* phage SCTP-2 with a genome size of 440,001 bp. According to the latest review on jumbo phages, the largest *Pseudomonas* phage is the *P. chlororaphis* phage 201φ2-1, with a 316,674 bp genome size (Thomas et al., 2008). All reported *Pseudomonas* jumbo phages are listed in Table 1.

**TABLE 1:** A full list of jumbo phages infecting *Pseudomonas* species, including phage MIJ3 (red).

				**Genome**
	**Accession**	**Phage name**	**Classification**	**length (bp)**
1	NC_010821	201phi2-1	Phikzvirus	316,674
2	HQ630627	PhiPA3	Phikzvirus	309,208
3	MF042360	Phabio	Phikzvirus	309,157
4	LR588166	MIJ3	Unclassified	288,170
5	MK797984	PA5oct	Unclassified	287,182
6	JN627160	OBP	Unclassified	284,757
7	NC_017972	Lu11	Unclassified	280,538
8	AF399011	phiKZ	Phikzvirus	280,334
9	MF805716	SL2	Phikzvirus	279,696
10	KU521356	KTN4	Phikzvirus	279,593
11	AP019418	PA02	Phikzvirus	279,095
12	MF063068	Noxifer	Noxifervirus	278,136
13	JX233784	PA7	Phikzvirus	266,743
14	KF147891	PaBG	Unclassified	258,139
15	AJ697969	EL	Elvirus	211,215

Because of their large genome size, jumbo phages encode many hypothetical proteins with unknown functions, in addition to encoding homologs of bacterial proteins, which might be an indication for phage–bacterial evolutionary events. Unlike other well-studied phages such as T4, jumbo phages lack recognizable modular genome characteristics that help to classify this group of phages. Phylogenetic analysis of the essential phage elements such as the major capsid proteins, terminases, and other proteins, revealed the presence of a new and phylogenetically related group within the jumbo phages, these have been termed the “Rak2-like viruses” (Šimoliūnas et al., 2013). This group encompasses eight phages: CBB, vB_CsaM_GAP32, BF, vB_KleM-Rak2, K64-1, 121Q, vB_Eco_slurp01, PBECO4, and Atu_ph07 (Attai et al., 2018). Most of the other characterized jumbo phages belong to different taxonomic families and orders, making it a challenging task to determine the similarities between them. Therefore, the terms “megaphages” and “jumbo phages” are umbrella terms which refer to the size of phages rather than an inherent set of qualities.

Jumbo phages exhibit characteristics that are rarely, or not at all observed in phages with smaller genomes. However, most *Pseudomonas* jumbo phages share some lifestyle features. In particular, it has been shown by Chaikeeratisak et al. (2017a) that the genomes of *Pseudomonas* jumbo phages (201phi2-1, phiKZ, and phiPA3) encode conserved proteins responsible for the formation of the phage nucleus, and tubulin spindle in the host bacterium during phage replication. A nucleus-like structure is formed in the cytoplasm of infected bacteria after phage DNA injection. It surrounds the phage DNA and separates it from the host cytoplasm, allowing the replication and transcription of phage DNA to occur inside the “phage nucleus” (Chaikeeratisak et al., 2017b). It has been suggested that the presence of such a nucleus-like structure is a conserved characteristic feature of all jumbo phages infecting *Pseudomonas* species (Chaikeeratisak et al., 2017a).

In this article, we report the isolation and characterization of a novel *P. aeruginosa* jumbo phage designated MIJ3 (vB_PaeM_MIJ3) with a genome size of 288,170 bp. We describe the MIJ3 phage morphology, pattern of infectivity, genomics characteristics, and proteomic identification of some of the major structural components. We also discuss how phage MIJ3 is a unique phage representing a novel phage species and genus.

## Materials and Methods

### Bacterial Strains and Culture Conditions

*Pseudomonas aeruginosa* strain PAO1 was used for phage isolation and propagation. Cells were cultivated in LB (Luria-Bertani) broth at 37°C with shaking at 120 RPM. The bacterial strains used for the phage MIJ3 host range analysis were 44 Liverpool Epidemic Strains (LES) (Winstanley et al., 2009), 12 strains isolated from patients with ventilator-associated pneumonia (VSP) at the Leicester Royal Infirmary (Freestone et al., 2012), a strain isolated from a COPD patient (Source: Dr. Christopher Turkington) and 2 lab strains *P. aeruginosa* PAO1 and PA14 (Table 2).

**TABLE 2:** A summary list of *P. aeruginosa* strains used in this study (a full detailed list of strains is presented in Supplementary Table 2).

**Strain**	**Source**	**References**
PAO1	Lab strain	Lab strain
PA14	Lab strain	Lab strain
LES strains 1–40	Cystic fibrosis patients	Winstanley et al., 2009
LES400	Cystic fibrosis patient	
LES431	Cystic fibrosis patient	
LESB58	Cystic fibrosis patient	
LESB65	Cystic fibrosis patient	
LRI strains 1–12	Ventilator-associated pneumonia patients	Freestone et al., 2012
COPD	COPD patient	Dr. Christopher Turkington

### Isolation of Phage MIJ3

Phage MIJ3 was isolated from a manure run-off in an equine livery yard, on which sheep are also kept, in Leicestershire. Due to the nature of the sample, a series of filtration steps were performed, starting initially with a 25 mm filter, down to filters with 0.22 μm pore sizes, this in order to remove bacteria. The filtered sample was screened for the presence of culturable phages using the plaque assay technique (Bonilla et al., 2016). Briefly, a mixture containing 300 μl of mid-log bacterial culture, 100 μl of the filtered sample, and 7 ml of LB top agar (0.7%) was poured on to a LB base plate and incubated overnight at 37°C to allow for the formation of phage plaques.

### Plaque Purification and Phage Propagation

A single plaque was picked and resuspended in 500 μl of SM buffer (5.8 g NaCl, 2.0 g MgSO_4_⋅7H_2_O, 50 ml 1 M Tris–HCl, pH 7.4, in 1 L of dH_2_O). After incubating for 60 min at 37°C, the solution was centrifuged, and the supernatant was filtered through 0.22 μm pore size filters. To produce a homogenous phage stock, seven rounds of single plaque purification were performed. Thereafter, the propagation of the phage was performed by preparing a plate lysate. The lysate was centrifuged, and the supernatant was filtered through a 0.22 μm pore size filter to remove bacterial cells (Bonilla et al., 2016). The titer of propagated phage was measured by the spot test as described in Mazzocco et al. (2009) with modifications. Briefly, phage lysate was serially diluted (12-fold) in SM buffer, and 10 μl of each dilution was spotted on a double-layer LB plate. The bottom layer contains 1% (w/v) LB agar and the top layer is composed of 0.5% (w/v) LB agar mixed with 100 μl of exponentially growing *P. aeruginosa* culture. The plates were all dried for 25 min in a laminar flow hood (ASTEC, MicroFlow) before it was incubated at 37°C for 18 h. The plaque forming unit (pfu/ml) was measured by counting the plaques in the dilution that have 3–30 countable plaques using the following calculation: Average number of plaque × 100 × reciprocal of dilution = pfu/ml.

### Transmission Electron Microscopy

Transmission electron microscopy analysis was performed according to the protocol of Ackermann (2009). Phage MIJ3 was negatively stained using 2% uranyl acetate on a carbon-coated grid, and visualized using the JEM 1400 transmission electron microscope (JEOL Co., Japan) with an accelerating voltage of 80 kV (Leicester, United Kingdom). Phage particle dimensions were measured using ImageJ version 1.49o^1^ in relation with the scale bar generated from the microscope.

### Adsorption Assay

To determine the time required for phage MIJ3 to attach to its host *P. aeruginosa* PAO1, an adsorption assay was performed according to the protocol mentioned by Kropinski (2009). Briefly, 0.95 ml of LB broth, and three drops of chloroform were added to 12 labeled tubes and placed on ice to chill for 10 min. A mid-log phase bacterial culture was diluted to 10 ml to an OD_600_ of 0.2. Two flasks (A and C) were filled with 9 ml of bacterial culture and LB broth, respectively, and placed in a shaking water bath (60 rpm) at 37°C. At 0 min, 1 ml of the warmed phage being tested at a titer of 1 × 10^5^ pfu/ml was added. Immediately, 1 ml of phage was added to the control flask (C). Aliquots of 0.05 ml from flask (A) were removed to the chilled labeled tubes every 1 min and mixed vigorously. Plaque assays were performed using test tubes containing 0.1 ml of chloroform in order to measure the phage titration.

### One-Step Growth Curve

To define the latent period and the average burst size of MIJ3, the one-step growth experiment was performed as described previously (Hyman and Abedon, 2009). Briefly, 9 ml of bacterial culture at an OD_600_ of 0.2, and 90 μl of phage MIJ3 at a concentration of 10^8^ (MOI of 0.01) were mixed and incubated for 6 min at 37°C. The mixture was centrifuged at 4,500 rpm for 5 min at 4°C to pellet the cells. The resultant pellet was washed with 5 ml of fresh LB broth and centrifuged again. The pellet was re-suspended in 10 ml of LB broth, and the mixture was incubated at 37°C. Aliquots of 1 ml of the mixture were collected at 15-min intervals for 2 h. Phages at each time points, including time point 0, were diluted and enumerated via the plaque assay. The pfu/ml was calculated and plotted against time. The period of latency was observed, and phage burst size was calculated from the plotted curve.

### Killing Assay

To assess the killing activity of phage MIJ3 on the host, *P. aeruginosa* PAO1 growing in LB was infected with MIJ3 in different MOIs as described in Chen et al. (2018). Briefly, 30 μl of phage at concentrations of 10^10^, 10^9^, and 10^8^ PFU/ml were mixed separately with 270 μl of mid-log bacteria at concentration of 10^8^ CFU/ml to make MOIs of 10, 1, and 0.1, respectively. The mixtures were incubated at 37°C and the bacterial growth was then monitored by measuring optical densities at OD_600_ for 3 h. Bacterial culture without phages was used as a control. The data were obtained from three independent experiments.

### Thermal and pH Stability of Phage MIJ3

The stability of phage MIJ3 in different temperatures was investigated. Aliquots of phage MIJ3 were incubated at 40, 50, 60, 70, 80, 90, and 100°C for 1 h before enumerating the surviving phages by spot test. Phage stability under different pH conditions was carried out according to Ahmadi et al. (2016). SM buffer was prepared with different pH values, ranging from 1 to 10 using either 1 M HCL or 1 M NaOH. MIJ3 at a concentration of 10^11^ was added to each pH solution, and the solutions were incubated for an hour before quantifying the number of phages in each solution.

### Host Range Analysis

To determine the host range of MIJ3, 10 μl of phage lysate was spotted on to a lawn of each of the tested *P. aeruginosa* strains isolated from patients with CF or COPD. The appearance of the spot was observed after an overnight incubation of the plates at 37°C, and scored as (1) for complete lysis, (2) for turbid and weak lysis, and (3) for no lysis. Strains marked as 1 or 2 were analyzed further by spot testing to confirm any plaque formation.

### Phage DNA Extraction

The DNA from phage MIJ3 was extracted by using the phenol–chloroform–isoamyl alcohol method as described in Nale et al. (2015). Phage lysate was treated with DNase and RNase to remove free DNA. To degrade the phage capsid, EDTA, proteinase K, and SDS were added to the lysate, and the mixture was incubated for an hour at 55°C. Phenol–chloroform–isoamyl alcohol (25:24:1) was added to the mixture at a 1:1 ratio and vortexed. The resulting sample was centrifuged at 21,000 × *g* for 15 min. The top aqueous layer was collected and treated with 1 volume of 3 M sodium acetate, and 2 volumes of ice-cold absolute ethanol, and then incubated for 3 h at −20°C and, centrifuged at 21,000 × *g* for 15 min. The DNA pellet was washed with 75% ethanol and re-suspended in upH_2_O. The DNA concentration was measured using the Qubit fluorometer (Thermo Scientific).

### Sequencing and Bioinformatics Analysis

Genomic libraries of phage MIJ3 DNA were prepared using Illumina’s TruSeq^®^ DNA Library Prep Kit (300 bp, FC-121-2003, Illumina), and whole genome sequencing was carried out using the MiSeq^®^ FGx system (Illumina). The resulting FASTQ files were assembled with Megahit version 1.2.1 (Li et al., 2015, 2016). The genome was sequenced to an average coverage depth of 35.38×. The resultant single phage contig was annotated with Prokka version 1.12, using the protein model databases HAMAP, VOG and Bacteria Viruses. In addition to this, manual gene prediction using all of the complete viral genomes available within the NCBI database and the European Nucleotide Archive (ENA) was performed (Seemann, 2014). Another method of annotation was performed using the Rapid Annotation using Subsystem Technology (RAST) pipeline as described previously (McNair et al., 2018).

An MIJ3 genome map was generated and visualized using Artemis version 17.0.1 (Carver et al., 2011). For phylogenetic analysis, amino acid sequences of phage core proteins such as the major capsid and terminase were checked for similarities in the NCBI database using BLASTp tool. Moreover, phages with homology to the amino acid sequences of these MIJ3 proteins were selected to generate phylogenetic trees using the MEGA7 software (Molecular Evolutionary Genetics Analysis) version 7.0 (Kumar et al., 2016). Selected amino acid sequences of phage proteins (major capsid protein and terminase) were aligned using ClustalW, and the resulting alignment file was used to create phylogenetic trees by the neighbor-joining method (Saitou and Nei, 1987). Genome comparison was analyzed using EasyFig version 2.2.3 (Sullivan et al., 2011).

To determine genome-to-genome distance of phage MIJ3 and other phages in the databases, VICTOR online tool was used^2^. All pairwise comparisons of the amino acid sequences were conducted using the Genome-BLAST Distance Phylogeny (GBDP) method (Meier-Kolthoff et al., 2013) under settings recommended for prokaryotic viruses (Meier-Kolthoff and Göker, 2017). The resulting intergenomic distances were used to infer a balanced minimum evolution tree with branch support via FASTME including SPR post-processing (Lefort et al., 2015). Branch support was inferred from 100 pseudo-bootstrap replicates each. Trees were rooted at the midpoint (Farris, 1972). Taxon boundaries at the species, genus, and family level were estimated with the OPTSIL program (Göker et al., 2009), the recommended clustering thresholds (Meier-Kolthoff and Göker, 2017), and an *F*-value (fraction of links required for cluster fusion) of 0.5 (Meier-Kolthoff et al., 2014).

### Accession Numbers

Reads for phage MIJ3 were deposited to the ENA at the European Bioinformatics Institute (EBI) under project accession number PRJEB32093. The taxon ID of MIJ3 is 2567864, and the phage genome was submitted under accession number LR588166.

### Phage Purification Using CIM^®^ Monoliths

Phage MIJ3 was purified using an anion-exchange chromatography column as described in Vandenheuvel et al. (2018) with some modifications. Briefly, 5 ml of the phage lysate (1.5 × 10^11^ pfu/ml) was dialyzed overnight at 4°C against a loading buffer (4 g MgSO_4_⋅6H_2_O, 50 ml of 1 M Tris–HCl, pH 7.5, ultra-pure H_2_O up to 1 L). The CIMmultus^TM^ QA-1 Advanced Composite Column was attached to the AKTA^TM^ FPLC^TM^ system before washing with the loading buffer. The elution buffer [116.9 g (2 M) NaCl, 4 g MgSO_4_⋅6H_2_O, 50 ml of 1 M Tris–HCl, pH 7.5, ultra-pure H_2_O up to 1 L] was added to the phage lysate, and the mixture was loaded on to the column. The fractions were analyzed using the UNICORN^TM^ and GraphPad Prism software. Phage titers in each fraction were determined by spot tests (Supplementary Figure 1).

### Proteomics Analysis

An aliquot of 25 μl purified phage was suspended in SDS loading buffer and heated for 5 min at 95°C. Then, proteins were separated using 12% SDS-PAGE gel, and stained using Coomassie stain. The major bands were extracted from the gel and analyzed by mass spectrometry (LC–MS/MS) at the Protein Nucleic Acid Chemistry Laboratory (PNACL, University of Leicester, United Kingdom), in accordance with Lavigne et al. (2009).

## Results

### Isolation of Phage MIJ3

In an attempt to isolate novel phages capable of infecting *P. aeruginosa*, we sampled water from mixed animal manure (horse and sheep) from an equine yard. A sample of the manure runoff water was collected and analyzed for the presence of phages capable of infecting *P. aeruginosa* using spot tests. Several phage plaques were observed on the *P. aeruginosa* PAO1 lawn. Plaques with distinct morphologies were selected for further studies. A small distinct plaque (approx. 0.5 mm in diameter) was purified via several rounds of plaque assays. The isolated phage was designated as vB_PaeM_MIJ3 (referred to herein as MIJ3), according to the phage naming criteria suggested by Adriaenssens and Brister (2017).

### Morphological Characteristics of Phage MIJ3

**FIGURE 1:**
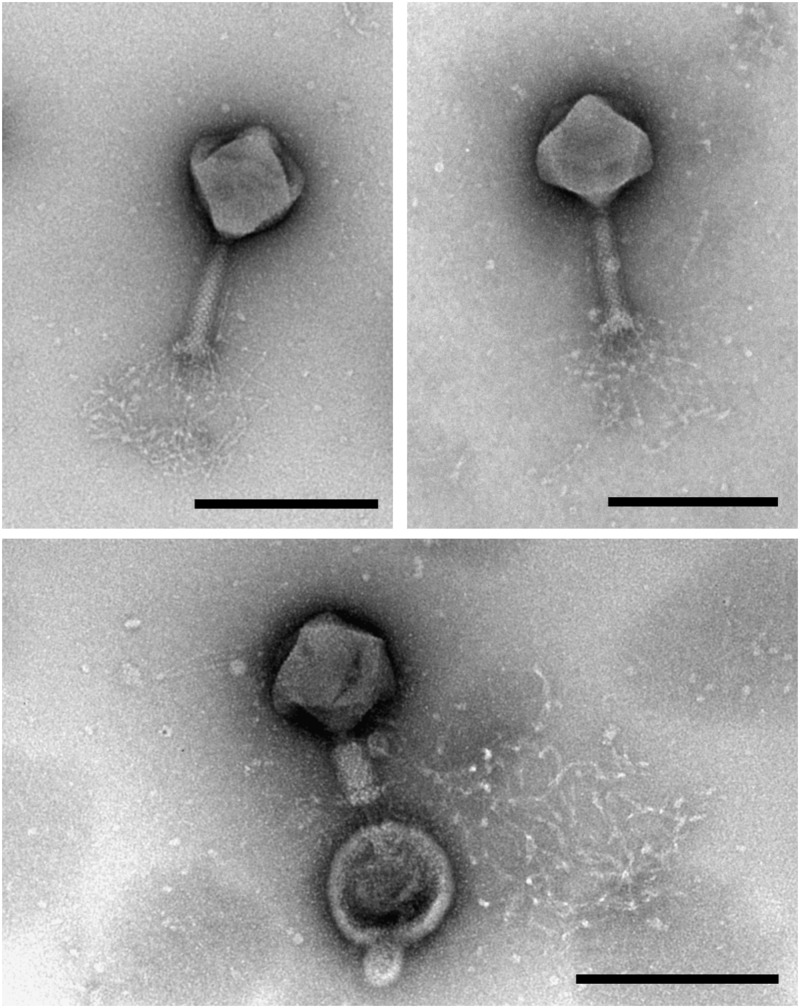
Transmission electron microscopy analysis of the purified phage MIJ3 revealed that MIJ3 belongs to the *Myoviridae* family and has an overall length of 258 nm. Phage MIJ3 possesses long tail fibers attached to the baseplate. Contracted tail can be observed upon interaction of MIJ3 with an outer membrane vesicle of *P. aeruginosa* (scale bar = 200 nm).

Transmission electron microscopy analysis of purified MIJ3 revealed that the phage belongs to the *Myoviridae* family. The average overall length of the phage from the top of the capsid to the base plate is 258 nm, with an average head height of 130 nm and 118 nm width. The phage tail has a height of 140 nm and length of 28 nm (*n* = 10 phages) (Figure 1). MIJ3 has the characteristic morphological features of a Myovirus morphotype A1: a classical phage head, a contractile tail with a collar, and a spiky baseplate attached to thin long kinked fibers (Figure 1).

### Adsorption Assay and One-Step Growth Curve

**FIGURE 2:**
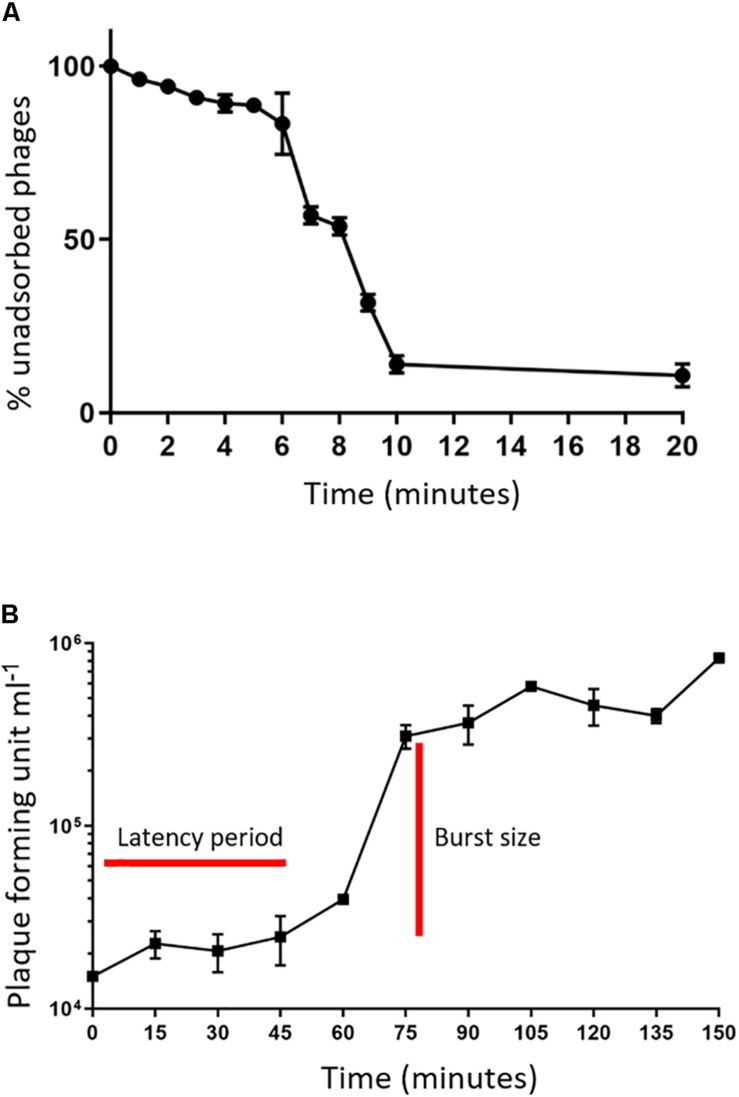
**(A)** Adsorption assay of phage MIJ3 on *P. aeruginosa*. **(B)** Growth parameters of MIJ3. The latent period of MIJ3 is between 45 and 50 min, and the burst size of one lytic cycle is 68 pfu per infected cell. Three biological replicates performed.

An adsorption assay was performed to identify the rate at which phage MIJ3 adsorbs to the surface of *P. aeruginosa* PAO1. The resulted graph (Figure 2A) revealed that around 50% of the phages attach to the host cells within 6 min, and 90% of the phages were adsorbed to host cells in 20 min. Phage growth parameters were determined by analyzing the phage growth cycle using the one-step growth curve experiment. The latent period of MIJ3 is between 45 and 50 min, and the burst size of one lytic cycle is approx. 68 pfu per infected cell (Figure 2B).

### Killing Assay

**FIGURE 3:**
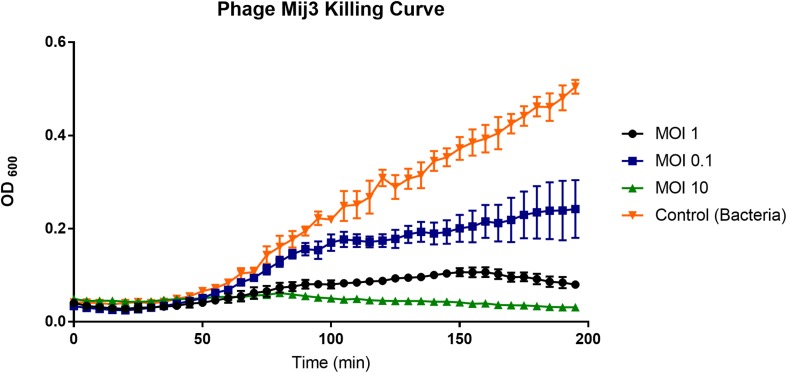
A graph showing the ability of phage MIJ3 to lyse *P. aeruginosa* PAO1 at different multiplicity of infections (0.1, 1, and 10) in LB medium at 37°C. At a high phage titer (MOI 10), bacterial growth was inhibited because of the phage infection (green). When an equal ratio of phage/bacteria was used (MOI 1), bacterial growth showed a slight increase after 90 min (black). At lower phage titer (MOI 0.1), bacteria were growing at higher levels but less than the non-infected cells (blue). The non-infected sample was used as a control (orange). Each experiment was done in triplicate, and OD_600_ values were measured, averaged, and plotted. Error bars represent SEM for three replicates.

To assess the killing activity of the phage on the host strain, *P. aeruginosa* PAO1 was grown in LB broth and infected with MIJ3 using different MOI values (10, 1, and 0.1), and the bacterial growth was monitored by measuring optical densities at OD_600_. In each case, the phage infection resulted in the inhibition of bacterial growth, which was progressively more prominent with an increase in the MOI. The lysis kinetics of MIJ3 was determined on strain PAO1. The optical density of the culture decreased at approximately 80 min post-infection with all three MOI values. At a high phage titer (MOI of 10), the bacterial growth rate was reduced because of phage infection. When an equal ratio of phage to bacteria was used (MOI of 1), bacterial growth showed a slight increase after 90 min. At a lower phage titer (MOI of 0.1), bacterial growth was higher than that observed with the cells infected at an MOI of 1 and 10, but still lower than the non-infected cells. The non-infected sample was used as a control, showing the growth of *P. aeruginosa* without adding any phage (Figure 3).

### Thermal and pH Stability of Phage MIJ3

**FIGURE 4:**
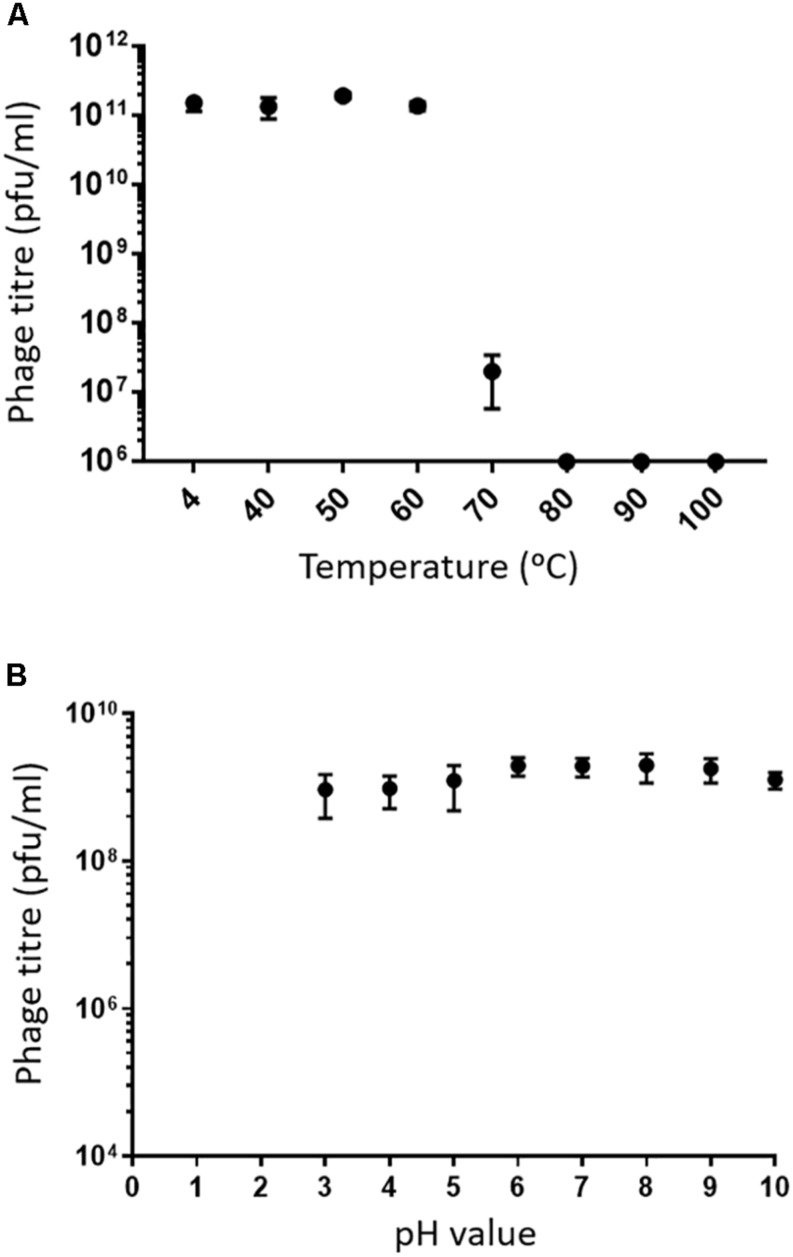
**(A)** Thermal stability of MIJ3: the phage was incubated in LB medium at different temperatures for 60 min. The phages survived at temperatures of 40, 50, and 60°C. At 70°C, phage concentration showed a 4-log drop. MIJ3 was not able to survive at 80, 90, and 100°C. Error bars represent SEM for three replicates. **(B)** pH stability of MIJ3. The phage is highly stable at pH values from 3 to 10; however, no survival was observed when phages at a pH of 2 and 1.

Assessing the stability of phages provides essential information for storage, transfer, and downstream experiments with the phage. Phage MIJ3 shows high stability at temperatures of 40, 50, and 60°C, as phage titers were similar to the control (4°C). After incubation for an hour at 70°C, there was a 4-log reduction in the phage MIJ3 titer, indicating that only 1 in 10,000 phages have survived under such conditions. Very few, if any, MIJ3 phage particles were able to survive at 80, 90, and 100°C (Figure 4A). Phage MIJ3 appears to be stable at pH values from 3 to 10. However, MIJ3 is not able to survive highly acidic pH values, as no phages survived testing against pH values of 2 and 1 (Figure 4B).

### Host Range of Phage MIJ3

The host range and lytic activity of MIJ3 on different clinical strains of *P. aeruginosa* was assessed by using spot test assays. Zones of lysis were observed and scored as (1) for complete lysis, (2) for turbid and weak lysis, and (3) for no lysis. For strains that were marked as (1) or (2), the phage stock was diluted and spotted on the bacterial lawn to show that the phage was forming single plaques, and the clearing was not due to lysis from without. The results reveal that MIJ3 is able to infect, and form plaques on, multiple clinical strains of *P. aeruginosa* isolated from patients with CF or VSP (as detailed in Supplementary Table 2).

### MIJ3 Genome and Phylogeny Analysis

The genome of phage MIJ3 was sequenced using the MiSeq platform, and the sequence was annotated by Prokka and RAST as described previously (Seemann, 2014; McNair et al., 2018). The phage was found to possess a large genome of 288,170 bp and was thus classified as a jumbo phage. The average G + C content of the genome of MIJ3 is 33.3%, which is in striking contrast to that of the bacterial host (66%). Blastn comparison of the MIJ3 sequence against the NCBI non-redundant database revealed that MIJ3 has significant similarity with one phage, a *Pseudomonas* phage vB_PaeM_PA5oct (98.72% identity). This was a surprising finding as the genome size of PA5oct was previously reported to be 375 kbp (Drulis-Kawa et al., 2014). However, the sequence deposited in the NCBI database revealed that the actual genome size of PA5oct is 287,182 bp (accession number: MK797984).

**FIGURE 5:**
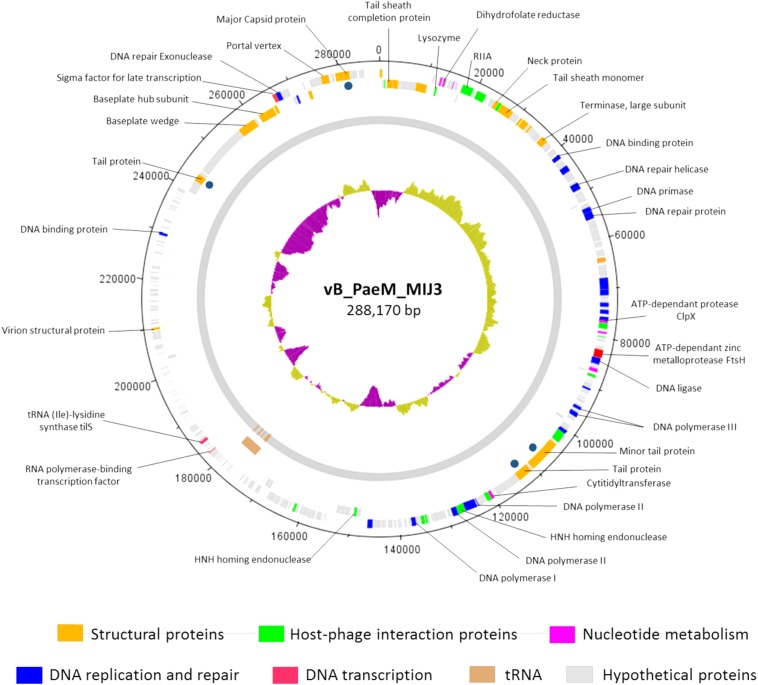
Circular genomic map of bacteriophage MIJ3. Phage MIJ3 possesses proteins involved in virion structure (orange), host–phage interaction (green), nucleotide metabolism (purple), DNA replication and repair (blue), and DNA transcription (pink). It also encodes host-like proteins such as FtsH (red) and tilS. There are 12 tRNAs in the genome of MIJ3 (brown). Other proteins with unknown functions are marked with gray. Dark blue dots indicate structural proteins confirmed by ESI-MS/MS. The map was generated using Artemis version 17.0.1.

The annotation of the phage MIJ3 sequence revealed the presence of 405 predicted open-reading frames (ORFs) and 12 tRNAs (Supplementary Table 3 and Figure 5). There are 21 ORFs encoding structural components, including 5 capsid proteins (gp41, gp406, gp409, gp410, and gp411) and 6 predicted tail proteins (gp1, gp4, gp36, gp142, gp144, and gp386). In addition, 24 proteins involved in DNA replication, recombination, repair, and transcription were identified. The majority of predicted genes (328 ORFs) encode hypothetical proteins with unknown functions. The MIJ3 genome encodes 12 tRNAs located between gp228 and gp246 and arranged as tRNA-Met, tRNA-Leu, tRNA-Asn, tRNA-Thr, tRNA-Met, tRNA-Leu, tRNA-Arg, tRNA-Met, tRNA-Pro, tRNA-Gly, tRNA-Ser, and tRNA-Ser.

**FIGURE 6:**
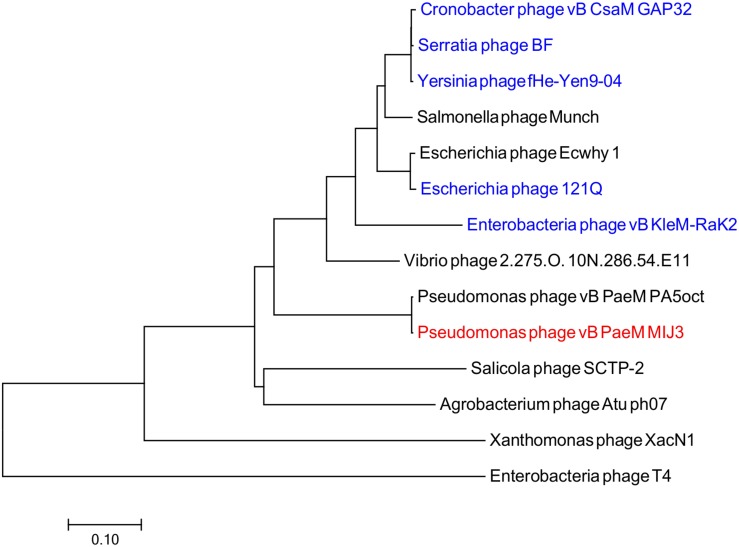
Phylogenetic tree comparing the major capsid protein of phage MIJ3 (red) with its corresponding in other jumbo phages, including Rak2-like phages (blue). Phages were selected as they share homology with the amino acid sequences of the major capsid using Blastp. Phage T4 was selected as an outgroup phage that is not closely related to the corresponding sequence of MIJ3. The evolutionary history was inferred using the neighbor-joining method and the tree was constructed using MEGA7.

**FIGURE 7:**
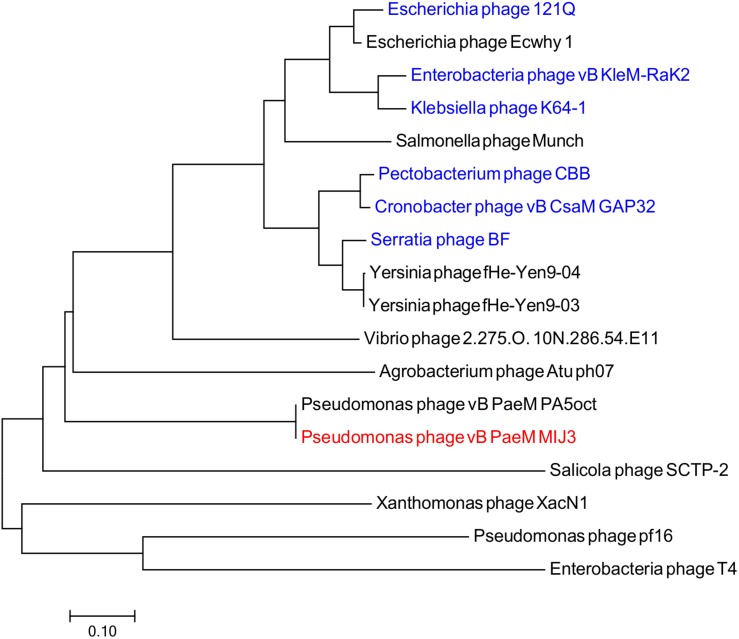
Phylogenetic tree comparing the terminase, large subunit of phage MIJ3 (red) with its corresponding in other jumbo phages, including Rak2-like phages (blue). Phages were selected as they share homology with the amino acid sequences of the terminase using Blastp. Phage T4 was selected as an outgroup phage that is not closely related to the corresponding sequence of MIJ3. The evolutionary history was inferred using the neighbor-joining method and the tree was constructed using MEGA7.

Phages MIJ3 and PA5oct have <10% ANI when compared with other phage sequences in the current databases, suggesting that they are a new species which belong to a new genus of phages that are not closely related to other phage groups. To find the location of MIJ3 in the phage continuum context, phylogenetic trees were generated for some of the predicted essential phage proteins, including the major capsid protein (Figure 6) and the terminase large subunit (Figure 7). From the assessment of phylogenetic trees, it seems that phage MIJ3 is not related to any other specific group of phages. The trees show the cluster of the newly identified group of jumbo phages called the Rak2-like phages family, which contains *Xanthomonas* phage XacN1 (Yoshikawa et al., 2018), *Agrobacterium* phage Atu_ph07 (Attai et al., 2018), *Serratia* phage BF (Casey et al., 2017), *Enterobacteria* phage vB_PcaM_CBB (Buttimer et al., 2017), *Cronobacter* phage vB_CsaM_GAP32 (Abbasifar et al., 2014), *Klebsiella* phage K64-1 (Pan et al., 2017), *Escherichia* phage 121Q (Ackermann and Nguyen, 1983), and *Klebsiella* phage vB_KleM-RaK2 (Šimoliūnas et al., 2013). Although the terminase of phage MIJ3 has similarities with RaK2-like phages, it is not considered as Rak2-like since all of these phages are over 300,000 bp, and due to the wide distance between the sequence of the major capsid protein sequence of phage MIJ3 and the Rak2-like phages. Furthermore, phylogenetic analysis of the major capsid protein tree shows that other *Pseudomonas* jumbo phages form an outgroup according to this protein sequence and are not closely related to MIJ3.

**FIGURE 8:**
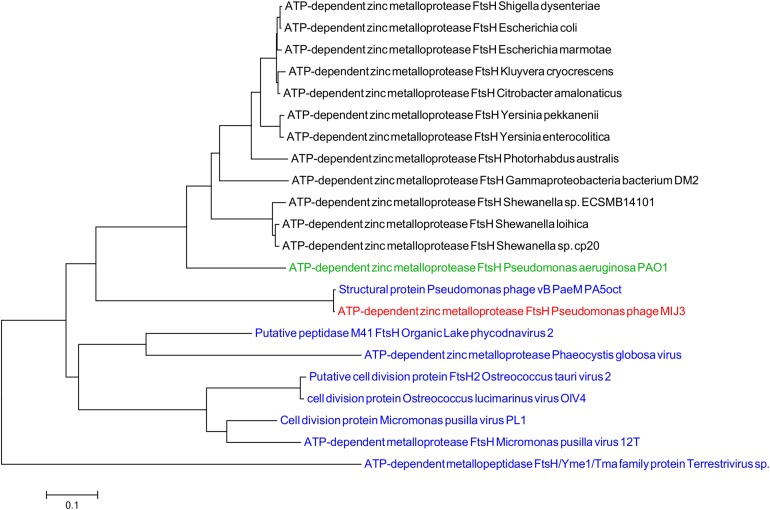
Phylogenetic tree of FtsH based on the amino acid sequence of phage MIJ3 and the most similar sequences in the database. It shows the FtsH of MIJ3 (red), the most similar amino acid sequences in viruses (blue), and bacteria (black) including *P. aeruginosa* PAOI, the bacterial host of MIJ3 (green).

Like other jumbo phages described to date, in addition to ORFs encoding “typical” phage proteins, the MIJ3 genome encodes for several proteins showing sequence similarity to bacterial host proteins. Several such “host-like” proteins found to be encoded in the MIJ3 genome are rare in phages, of which, two proteins not been found in any phages to date. These two proteins are homologs of the FtsH protein (ATP-dependent zinc metalloprotease) and TilS [tRNA(Ile)-lysidine synthase]. FtsH and TilS are widely conserved proteins in bacteria. FtsH, formerly known as HflB, plays an important role in maintaining membrane integrity, cell division, and in the heat shock response (Bieniossek et al., 2006). TilS is a tRNA modification enzyme and is essential for bacterial viability (Suzuki and Miyauchi, 2010). To analyze the evolutionary relationship of the unique phage borne FtsH in phage MIJ3, and to track down the origin of this protease, a phylogenetic tree of FtsH was created as described previously (Figure 8). The analysis shows that the bacterial FtsH proteins are grouped separately from viral FtsH. The FtsH of phage MIJ3 has a closer relationship to the bacterial FtsH, rather than the viral FtsH.

**FIGURE 9:**
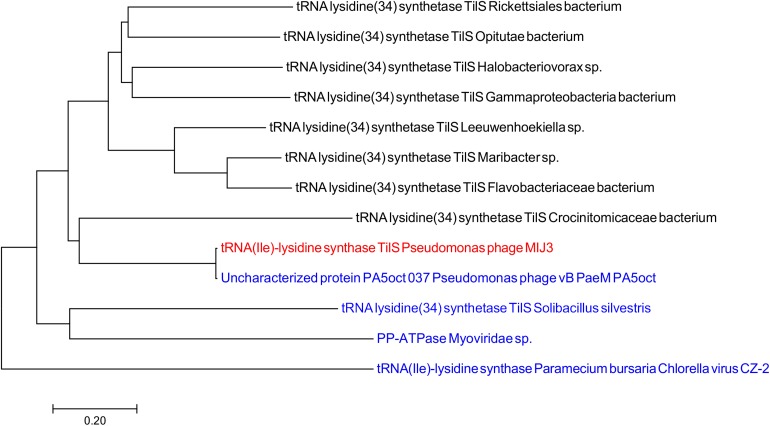
Phylogenetic tree of TilS based on the amino acid sequence of phage MIJ3 and the most similar sequences in the database. It shows the TilS of MIJ3 (red), the most similar TilS amino acid sequences in viruses (blue), and bacteria (black).

For the same purpose, a phylogenetic tree for the TilS protein was constructed using the neighbor-joining method shows that TilS MIJ3 amino acid sequence is identical to the sequence of the uncharacterized protein in phage PA5oct. Similar to the findings from the FtsH phylogeny analysis, the TilS sequence of phage MIJ3 is a closer relative to the bacterial TilS protein rather than the viral TilS. Although the genome of *P. aeruginosa* PAO1, the bacterial host of MIJ3, encodes TilS protein, the Tils protein of MIJ3 is distantly related to the host Tils (Figure 9).

**FIGURE 10:**
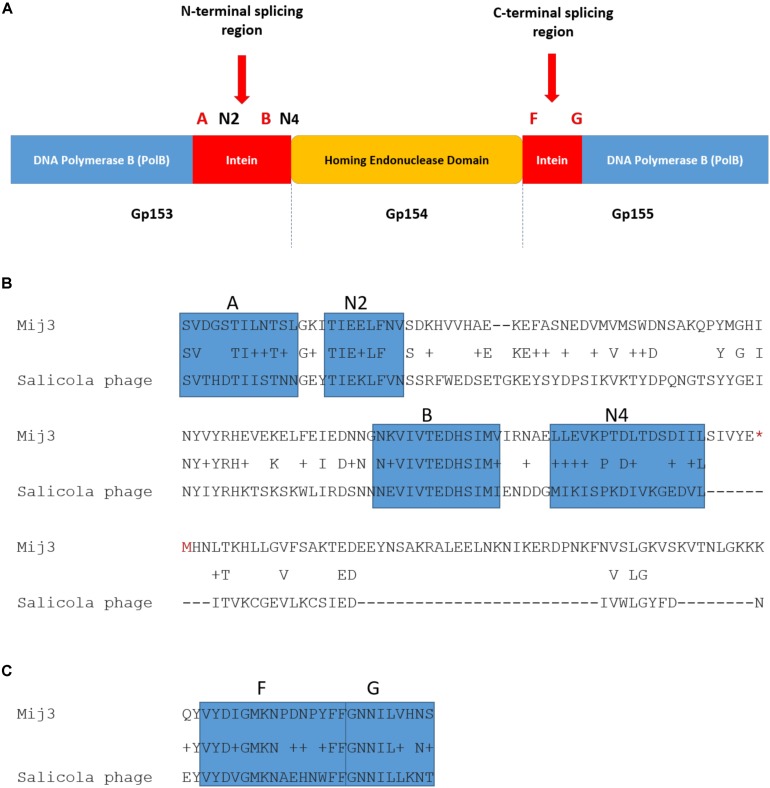
The presence of intein in MIJ3. **(A)** A diagram illustrating the location of the intein and its conserved motifs in the genome of MIJ3 jumbo phage. **(B)** N-terminal splicing region sequence alignment between inteins inserted in the PolB of MIJ3 and the *Salicola* phage SCTP-2 with the identified intein motifs highlighted as blocks A, N2, B, and N4. **(C)** C-terminal splicing region sequence alignment of the intein in both phages with the identified intein motifs are highlighted as blocks F and G.

Further analysis of the annotated MIJ3’s genomic data revealed another unusual feature: a putative split intein with an HNH homing endonuclease family (orf154) within the genes encoding the DNA polymerase B (orfs153 and 155) (Figure 10). Inteins are mobile self-splicing elements intervening the host protein sequence, and they are excised post-translation through a process known as protein splicing (Perler, 2005). They are often associated with the homing endonuclease domain to mediate this genetic transfer (Dassa et al., 2009). Intein motifs can be identified by conserved residues that are involved in protein splicing (Pietrokovski, 1994; Perler et al., 1997). In phage MIJ3, the intein motif blocks were identified in the N-terminal splicing region: block A (SVDGSTILNTSL), block N2 (TIEELFNV), block B (NKVIVTEDHSIMV), and block N4 (LLEVKPTDLTDSDIIL); and in the C-terminal splicing region: block F (VYDIGMKNPDNPYFF) and block G (GNNILVHNS). Bioinformatic analysis of the protein-splicing sequence element of the phage revealed that the putative MIJ3 split intein shares a similarity with the mini-intein present in the gene encoding PolB in another jumbo phage, the *Salicola* phage SCTP-2 (Figure 10).

### Phage Purification Using CIM^®^ Monoliths and Proteomics Analysis

**FIGURE 11:**
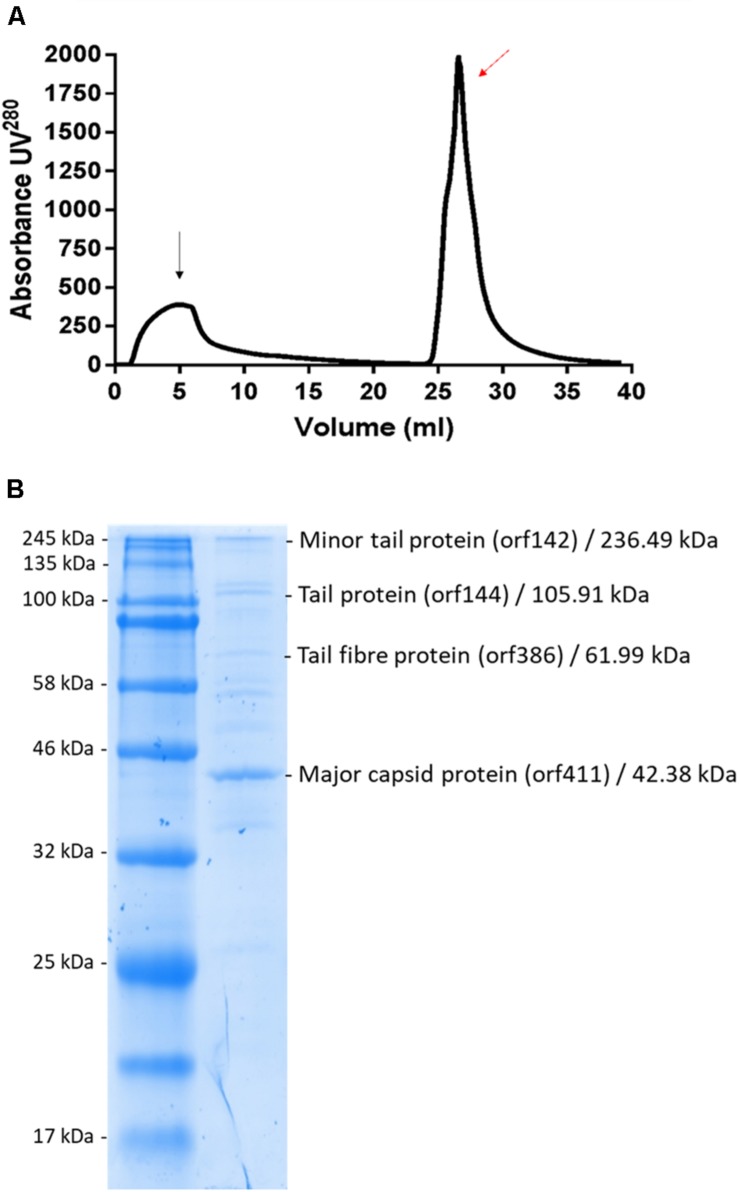
**(A)** Linear gradient of MIJ3 purification using a DEAE column. The black arrow indicates the flow-through and the red arrow indicates the peak that corresponds with the presence of phage MIJ3. **(B)** Proteomic analysis of phage MIJ3 particles. Proteins of purified phage particles **(B)** were separated in 12% (wt/vol) polyacrylamide gel by SDS-PAGE and stained with Coomassie brilliant blue. The protein bands were subjected to LC–MS/MS analysis. On the right are the descriptions of the genes, their deduced molecular sizes based on the ORF sequences, and their possible functions. Positions of size markers are shown on the left **(A)**.

Phage MIJ3 was purified by using an anion-exchange chromatography column as previously described in the section “Materials and Methods” (Figure 11A). Eluted fragments under the phage peak were pooled, and the phage titers were calculated using spot tests. The purified MIJ3 titer was 4 × 10^10^ pfu/ml, indicating that the phage titer decreased by 1 log, compared to the titer of the non-purified phage, which was 1.2 × 10^11^ pfu/ml. The purified MIJ3 virions were separated by SDS-PAGE, and several major protein bands were analyzed by MS. Four structural proteins were identified by LC–MS/MS, including the major (gp144) and the minor tail proteins (gp142), a tail fiber protein (gp386), and the major capsid protein (gp411) (Figure 11B). These proteins were confirmed by the genome annotation of the phage in relation to the sizes of their amino acid sequences in the gel and in the annotation information.

## Discussion

Phages are abundant in different environments but very large phages are not commonly isolated. They could be removed during the filtration process due to their large virion sizes, or because of their limited mobility on semi-solid plates (Serwer et al., 2007). Genomes of jumbo phages and megaphages encode a number of hypothetical proteins with unknown functions, and their biological features are understudied and not found in small phages. Jumbo phages have more genes encoding DNA replication and metabolism such as DNA polymerase and RNA polymerase in addition to genes responsible for translation (Yuan and Gao, 2017). Abundance of these genes indicates less dependency on the host in addition to wider host ranges. More studies are expected to be conducted to reveal more features of these phages.

Phage MIJ3, described here, is an interesting representative of jumbo phages for numerous reasons. The phage is effective against clinical strains of *P. aeruginosa* that were isolated from patients with CF or COPD. This suggests that MIJ3 can potentially be used for further phage therapy trials, especially for infections with multi-drug resistant *P. aeruginosa*. It can also be added to the existing phage cocktails for more effective outcomes. There is the need to test phage MIJ3 with other *P. aeruginosa* phages against more strains to expand the therapeutic applicability of jumbo phages. Other jumbo phages such as phiPA3, PA5oct, phiKZ, SL2, KTN4, PA02, PA7, and PaBG also have the ability to infect a wide range of clinical isolates of *P. aeruginosa* (Mesyanzhinov et al., 2002; Monson et al., 2011; Sykilinda et al., 2014; Latz et al., 2017). These phages were isolated from different sources all of which are, respectively, different to each other, examples include sewage, mudflat, lake water, and manure, indicating the diversity of available sources of phages with promising therapeutic potential.

*Pseudomonas aeruginosa* strains are associated with mucus formations and are able to surround themselves with exopolysaccharides, which are alginic acids, providing resistance to antibiotics and immune cells, especially in CF patients (Glonti et al., 2010; Morello et al., 2011). Phages have showed promising effects in treating infections caused by multi-drug resistant *P. aeruginosa* isolated from patients with CF *in vivo* and *in vitro* (Furfaro et al., 2018). Therefore, phage MIJ3 can potentially be used in phage therapy against *Pseudomonas* infections. In addition, phage endolysins and peptidoglycan-degrading enzymes from phages phiKZ and EL have been previously tested as potential antibacterial agents against *Pseudomonas* infections (Briers et al., 2007). Similar proteins from MIJ3 could be investigated and used for the same purpose.

The physical and chemical stability of phage MIJ3 was determined to provide more information with regards to phage storage, transport, and potential applications. MIJ3 is stable at temperatures ranging from 4 to 60°C; however, only approximately 1 in 10,000 phage particles survived when incubated at 70°C for 1 h, and it was inactivated when incubated at 80, 90, and 100°C. More than 90% of MIJ3 phage survived at pH values ranging from pH 10 to 3 but, couldn’t survive at pH 1 and 2. It has been suggested that tailed phages are more resistant to adverse conditions such as temperature change and pH (Ackermann et al., 2004). For therapeutic purposes, phages are required to maintain stability in a wide range of temperatures and pH, to resist changes in environmental and ambient condition, and MIJ3 appears to satisfy these criteria. The ability to withstand the aforementioned conditions provides valuable information as to whether MIJ3 would be able to survive downstream processing steps such as encapsulation or spray drying, especially if MIJ3 was to be used for therapeutic purposes.

The genomic sequence of MIJ3 and PA5oct have greater than 95% suggesting that they represent a new phage species according to the main species demarcation criterion (Adriaenssens and Brister, 2017). These phages are unique as their genome sequences share ANI of < 1% with other phages in the database. Members of the same genus share a high degree of nucleic acid similarity (>50%) with others, along with factors such as genome length, average and percentage of coding sequencing, number of tRNA, and the presence of certain signature genes in member viruses (Adriaenssens and Brister, 2017).

**FIGURE 12:**
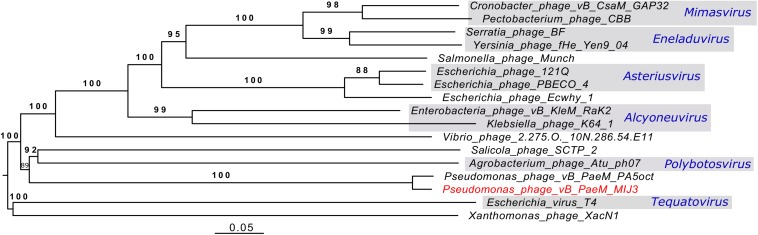
A phylogenetic tree of phage MIJ3 and the closest phages. It shows the phylogenomic GBDP tree yielding average support of 97%. The numbers above branches are GBDP pseudo-bootstrap support values from 100 replications. The branch lengths of the resulting VICTOR trees are scaled in terms of the respective distance formula used. Phage MIJ3 is marked in red color, and genus of classified phages is marked in blue. The tree was generated using the VICTOR server.

Analysis of the genome-to-genome phylogeny of phage MIJ3 and other phages (Figure 12), and the phylogenetic trees of the major capsid protein and terminase (large subunit) (Figures 6, 7), shows that MIJ3 has a distant relation with other phages including GAP32, K64-1, Rak2, PBECO 4, and 121Q. According to the ICTV database, these phages are classified into different genera. Phages K64-1 and Rak2 are members of the *Alcyoneusvirus* genus, phages 121Q and PBECO4 belong to the *Asteriusvirus* genus, and phage GAP32 belongs to the *Mimasvirus* genus. In addition to VICTOR analysis, this suggests that MIJ3 and PA5oct represent a new genus.

The phage MIJ3 genome encodes proteins that have not yet been reported in phages, including the ATP-dependent zinc metalloprotease, FtsH (gp107), and the tRNA (Ile)-lysidine synthase, TilS (gp263). The functions of these proteins in MIJ3 are unknown. In bacteria, both FtsH and TilS play important roles and are essential to the viability of the cell (Shotland et al., 2000; Soma et al., 2003; Lin et al., 2013). Therefore, it is tempting to speculate that their role in MIJ3 could be to maintain the host viability during phage replication. This can be examined by constructing FtsH and or TilS mutants and comparing the infectivity of these mutants with the wild-type phage. The amino acid sequences of *ftsH* and *tilS* in MIJ3 are homologous to gp342 and gp37, respectively, of phage PA5oct, which were annotated as a structural protein and an uncharacterized protein, respectively. Further research is needed to confirm the nature and the function of these unique phage genes and proteins in MIJ3 and PA5oct.

**FIGURE 13:**
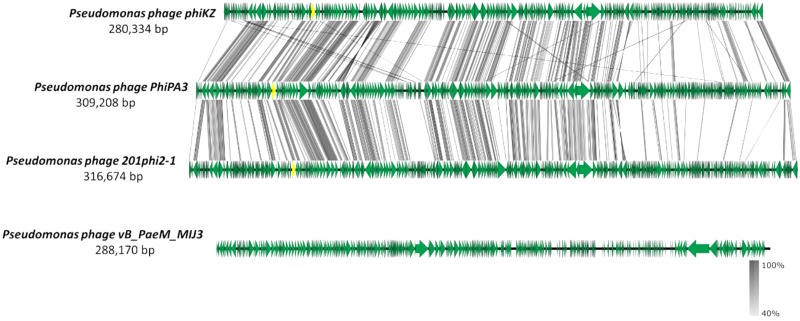
Comparative analysis of phage MIJ3 genome with the genomes of other jumbo *Pseudomonas* phages phiKZ, PhiPA3, and 201phi2-1. Phage MIJ3 shows no homology to the other three phages. The yellow areas indicate the conserved genes encoding the phage nucleus and spindle tubulin in phages phiKZ, PhiPA3, and 201phi2-1, of which is absent in phage MIJ3.

The MIJ3 jumbo phage has several unusual genomic features, such as the GC content being drastically different from the host genome, or the presence of rare phage genes. However, MIJ3 is also unusual due to the absence of some features that were believed to be conserved across all *Pseudomonas* jumbo phages, such as the phage nucleus and phage spindle (Chaikeeratisak et al., 2017a). During the replication step, *Pseudomonas* phages 201phi2-1, phiPA3, and phiKZ can form a proteinaceous nucleus, or shell inside the host. This nucleus encloses viral DNA and enzymes involved in the DNA replication and transcription. Unlike these *Pseudomonas* jumbo phages, phage MIJ3 does not appear to encode the PhuZ bipolar tubulin spindle, as genome comparison between MIJ3 and phages 201φ2-1, phiPA3, and φKZ revealed that there is no gene encoding a protein with recognizable sequence similarity to PhuZ in the sequence of phage MIJ3 (Figure 13).

MIJ3 DNA sequence is highly similar to that of another *Pseudomonas* phage, PA5oct (Accession Number MK797984). An earlier publication describing PA5oct suggests that its genome size is approximately 375 kb (Drulis-Kawa et al., 2014). However, the genomic sequence of phage PA5oct was released in April 2019, and is in fact 287,182 bp. It is intriguing that two highly related phages were independently isolated from two geographically distant locations: phage MIJ3 (described here) was isolated from a manure sample of a domestic farm near Leicester, United Kingdom, while phage PA5oct was isolated from a sewage sample from an irrigated field near Wrocław, Poland (Drulis-Kawa et al., 2014). These findings not only suggest a wide distribution of MIJ3/PA5oct phages, but also indicate their high genomic stability.

## Conclusion

In conclusion, this study presents analyses of the biological and genomic features of MIJ3, suggesting that phage MIJ3 and the previously identified phage PA5oct should be grouped together into a novel phage genus. In addition, phage MIJ3 has antimicrobial activity against clinical *P. aeruginosa* isolates, suggesting the potential use of this phage as a therapeutic agent.

## Data Availability Statement

Whole genome sequencing datasets generated for this study can be found in the ENA, LR588166.

## Author Contributions

MI carried out the main body of research, performed the bioinformatics analysis, and wrote the manuscript. BA contributed to the phage growth experiments. FP contributed to the pH stability and adsorption assay experiments. AD contributed to the AKTA purification work. NB contributed to the sequencing. AM edited the manuscript and contributed in bioinformatics analysis. MC edited the manuscript and collected the sample for phage isolation. EG supervised the work progress and edited the manuscript.

## Conflict of Interest

The authors declare that the research was conducted in the absence of any commercial or financial relationships that could be construed as a potential conflict of interest.

## Footnotes

1 https://imagej.nih.gov/ij/

2 https://ggdc.dsmz.de/victor.php
