# Supplementary material for: vB_PaeM_MIJ3, a Novel Jumbo Phage Infecting Pseudomonas aeruginosa, Possesses Unusual Genomic Features
Source: Front Microbiol. 2019 Nov 28;10:2772. doi: 10.3389/fmicb.2019.02772 (PMC6892783; doi:10.3389/fmicb.2019.02772)
Supplement: Supplementary file 1 [file Data_Sheet_1.docx]

Supplementary Material

vB_PaeM_MIJ3, a Novel Jumbo Phage Infecting *Pseudomonas aeruginosa,* Possesses Unusual Genomic Features

Mohammed Imam, Bandar Alrashid, Faizal Patel, Ahmed Dowah, Nathan Brown, Andrew Millard, Martha R.J. Clokie and Edouard E. Galyov^*^

*** Correspondence:** Edouard E. Galyov: eg98@leicester.ac.uk

# Supplementary Table 1: A full list of megaphages (Blue) and jumbo phages. MIJ3 is typed with red colour.

| **Accession** | **Description** | **Classification** | **Genome Length(bp)** |
| --- | --- | --- | --- |
| MK250027 | Prevotella phage Lak-B8 | unclassified | 551,627 |
| MK250026 | Prevotella phage Lak-B7 | unclassified | 550,702 |
| MK250023 | Prevotella phage Lak-B4 | unclassified | 550,552 |
| MK250028 | Prevotella phage Lak-B9 | unclassified | 550,053 |
| MK250021 | Prevotella phage Lak-B2 | unclassified | 549,839 |
| MK250020 | Prevotella phage Lak-B1 | unclassified | 547,991 |
| MK250022 | Prevotella phage Lak-B3 | unclassified | 546,746 |
| MK250025 | Prevotella phage Lak-B6 | unclassified | 546,689 |
| MK250024 | Prevotella phage Lak-B5 | unclassified | 543,529 |
| MK250016 | Prevotella phage Lak-A1-II | unclassified | 541,664 |
| MK250017 | Prevotella phage Lak-A1-III | unclassified | 541,664 |
| MK250018 | Prevotella phage Lak-A1-IV | unclassified | 541,664 |
| MK250015 | Prevotella phage Lak-A1-I | unclassified | 541,643 |
| MK250019.1 | Prevotella phage Lak-A2 | unclassified | 541,299 |
| MK250029 | Prevotella phage Lak-C1 | unclassified | 540,217 |
| NC_023719 | Bacillus virus G | Myoviridae | 497,513 |
| MF403008 | Agrobacterium phage Atu_ph07 | Myoviridae | 490,380 |
| MF360958 | Salicola phage SCTP-2 | Myoviridae | 440,001 |
| MH494197 | Escherichia phage CMSTMSU | Myoviridae | 386,442 |
| AP018399 | Xanthomonas phage XacN1 | Myoviridae | 384,670 |
| KU574722 | Pectobacterium phage CBB | Myoviridae | 378,379 |
| NC_019401 | Cronobacter phage vB_CsaM_GAP32 | Myoviridae | 358,663 |
| KY630187 | Serratia phage BF | Myoviridae | 357,154 |
| MH791411 | Escherichia phage Ecwhy_1 | unclassified | 354,537 |
| LT960551 | Yersinia phage fHe-Yen9-04 | Myoviridae | 354,378 |
| MH383160 | Escherichia phage UB | Myoviridae | 353,081 |
| LT960552 | Yersinia phage fHe-Yen9-03 | Myoviridae | 352,596 |
| MK268344 | Salmonella phage Munch | unclassified | 350,103 |
| MG592671 | Vibrio phage 2.275.O._10N.286.54.E11 | unclassified | 348,911 |
| NC_025447 | Escherichia phage 121Q | Myoviridae | 348,532 |
| KC295538 | Escherichia phage PBECO 4 | Myoviridae | 348,113 |
| LT603033 | Escherichia phage vB_Eco_slurp01 | unclassified | 348,043 |
| NC_027399 | Klebsiella phage K64-1 | Myoviridae | 346,602 |
| JQ513383 | Enterobacteria phage vB_KleM-RaK2 | Myoviridae | 345,809 |
| MH588546 | Caulobacter phage CcrBL9 | Siphoviridae | 322,272 |
| MH588547 | Caulobacter phage CcrSC | Siphoviridae | 317,488 |
| **NC_010821** | **Pseudomonas phage 201phi2-1** | Myoviridae | **316,674** |
| **HQ630627** | **Pseudomonas phage PhiPA3** | Myoviridae | **309,208** |
| **MF042360** | **Pseudomonas phage Phabio** | Myoviridae | **309,157** |
| MH588545 | Caulobacter phage CcrPW | Siphoviridae | 308,141 |
| MH595538 | Vibrio phage BONAISHI | Myoviridae | 288,967 |
| **LR588166** | **Pseudomonas phage Mij3** | **Myoviridae** | **288,170** |
| **JN627160** | **Pseudomonas phage OBP** | Myoviridae | **284,757** |
| **NC_017972** | **Pseudomonas phage Lu11** | Myoviridae | **280,538** |
| **AF399011** | **Pseudomonas virus phiKZ** | Myoviridae | **280,334** |
| NC_019406 | Caulobacter phage CcrColossus | Siphoviridae | 279,967 |
| AP017924 | Ralstonia phage RP12 | Myoviridae | 279,845 |
| **MF805716** | **Pseudomonas phage SL2** | Myoviridae | **279,696** |
| **KU521356** | **Pseudomonas phage KTN4** | Myoviridae | **279,593** |
| **AP019418** | **Pseudomonas phage PA02** | Myoviridae | **279,095** |
| **MF063068** | **Pseudomonas phage Noxifer** | Myoviridae | **278,136** |
| AP017925 | Ralstonia phage RP31 | Myoviridae | 276,958 |
| MF285618 | Serratia phage 2050HW | Myoviridae | 276,025 |
| MG655269 | Erwinia phage vB_EamM_MadMel | Myoviridae | 275,000 |
| MG655270 | Erwinia phage vB_EamM_Mortimer | Myoviridae | 273,914 |
| KU886225 | Erwinia phage vB_EamM_Deimos-Minion | Myoviridae | 273,501 |
| KU886222 | Erwinia phage vB_EamM_Special G | Myoviridae | 273,224 |
| MG655268 | Erwinia phage vB_EamM_Desertfox | Myoviridae | 272,458 |
| MG655267 | Erwinia phage vB_EamM_Bosolaphorus | Myoviridae | 272,228 |
| KU886224 | Erwinia phage vB_EamM_RAY | Myoviridae | 271,182 |
| KU886223 | Erwinia phage vB_EamM_Simmy50 | Myoviridae | 271,088 |
| KF806589 | Erwinia phage Ea35-70 | Myoviridae | 271,084 |
| **JX233784** | **Pseudomonas phage PA7** | Myoviridae | **266,743** |
| MH248138 | Erwinia phage vB_EamM_Alexandra | Myoviridae | 266,532 |
| AJ972879 | Yersinia phage phiR1-37 | Myoviridae | 262,391 |
| MH460463 | Dickeya phage vB_DsoM_AD1 | Myoviridae | 261,658 |
| KY984068 | Erwinia phage vB_EamM_Y3 | Myoviridae | 261,365 |
| KY448244 | Erwinia phage vB_EamM_Yoloswag | Myoviridae | 259,700 |
| **KF147891** | **Pseudomonas phage PaBG** | Myoviridae | **258,139** |
| LT598654 | Phage NCTB | Myoviridae | 257,877 |
| KT895374 | Bacillus phage vB_BpuM-BpSp | Myoviridae | 255,569 |
| MH389777 | Dickeya phage vB_DsoM_JA11 | Myoviridae | 255,356 |
| MH460462 | Dickeya phage vB_DsoM_JA33 | Myoviridae | 255,356 |
| MH460460 | Dickeya phage vB_DsoM_JA13 | Myoviridae | 254,061 |
| MH460461 | Dickeya phage vB_DsoM_JA29 | Myoviridae | 253,323 |
| GU071092 | Prochlorococcus phage P-SSM2 | Myoviridae | 252,407 |
| MF360957 | Bacillus virus PBS1 | Myoviridae | 252,197 |
| HQ632825 | Prochlorococcus phage P-SSM5 | Myoviridae | 252,013 |
| KU878088 | Bacillus phage AR9 | Myoviridae | 251,042 |
| MH221129 | Salmonella phage SPAsTU | Myoviridae | 250,739 |
| KT919973 | Vibrio phage phi-ST2 | Myoviridae | 250,485 |
| KT919972 | Vibrio phage phi-Grn1 | Myoviridae | 248,605 |
| NC_028829 | Vibrio phage ValKK3 | Myoviridae | 248,088 |
| HQ317393 | Vibrio phage nt-1 | Myoviridae | 247,511 |
| KC131129 | Vibrio phage VH7D | Myoviridae | 246,964 |
| JN849462 | Vibriophage phi-pp2 | Myoviridae | 246,421 |
| NC_031010 | Erwinia phage vB_EamM_Kwan | Myoviridae | 246,390 |
| KX397364 | Erwinia phage vB_EamM_Asesino | Myoviridae | 246,290 |
| MH426724 | Erwinia phage Wellington | Myoviridae | 244,950 |
| NC_031126 | Erwinia phage vB_EamM_ChrisDB | Myoviridae | 244,840 |
| AY283928 | Vibrio phage KVP40 | Myoviridae | 244,834 |
| KX397373 | Erwinia phage vB_EamM_Stratton | Myoviridae | 243,953 |
| NC_019929 | Erwinia phage phiEaH2 | Myoviridae | 243,050 |
| KX397370 | Erwinia phage vB_EamM_Machina | Myoviridae | 241,654 |
| NC_031120 | Erwinia phage vB_EamM_Caitlin | Myoviridae | 241,147 |
| KX397371 | Erwinia phage vB_EamM_Parshik | Myoviridae | 241,050 |
| KX397368 | Erwinia phage vB_EamM_Huxley | Myoviridae | 240,761 |
| NC_027402 | Salmonella phage SPN3US | Myoviridae | 240,413 |
| KU726251 | Enterobacteria phage SEGD1 | Myoviridae | 239,461 |
| MG592456 | Vibrio phage 1.081.O._10N.286.52.C2 | unclassified | 239,318 |
| AP017972 | Vibrio phage pTD1 | Myoviridae | 239,276 |
| MG720308 | Vibrio phage Aphrodite1 | Myoviridae | 237,722 |
| KY290955 | Aeromonas phage 65.2 | Myoviridae | 236,567 |
| KC131130 | Vibrio phage VP4B | Myoviridae | 236,053 |
| MF459647 | Erwinia phage vB_EamM_Joad | Myoviridae | 235,374 |
| NC_015251 | Aeromonas virus 65 | Myoviridae | 235,229 |
| MF459646 | Erwinia phage vB_EamM_RisingSun | Myoviridae | 235,108 |
| KU935715 | Acinetobacter phage vB_AbaM_ME3 | Myoviridae | 234,900 |
| LC168164 | Tenacibaculum phage pT24 | Siphoviridae | 234,670 |
| NC_005260 | Aeromonas virus Aeh1 | Myoviridae | 233,234 |
| HQ317391 | Cyanophage S-SSM6a | Myoviridae | 232,883 |
| GU071098 | Synechococcus phage S-SSM7 | Myoviridae | 232,878 |
| KY499642 | Vibrio phage pVa-21 | Myoviridae | 231,998 |
| MH791404 | Aeromonas phage Assk | unclassified | 231,839 |
| NC_019538 | Aeromonas phage CC2 | Myoviridae | 231,743 |
| AB366653 | Ralstonia phage phiRSL1 | Myoviridae | 231,255 |
| MF498774 | Aeromonas phage AS-yj | Myoviridae | 230,183 |
| MF498775 | Aeromonas phage AS-sw | Myoviridae | 230,024 |
| MF498773 | Aeromonas phage AS-szw | Myoviridae | 229,957 |
| MF448340 | Aeromonas phage AS-zj | Myoviridae | 229,929 |
| MH791414 | Aeromonas phage Aswh_1 | unclassified | 229,730 |
| KX397372 | Erwinia phage vB_EamM_Phobos | Myoviridae | 229,501 |
| KY555145 | Caulobacter phage Ccr29 | Siphoviridae | 229,319 |
| MH791398 | Aeromonas phage Asswx_1 | unclassified | 229,103 |
| NC_026927 | Synechococcus phage ACG-2014f | Myoviridae | 228,143 |
| NC_014636 | Aeromonas phage phiAS5 | Myoviridae | 225,268 |
| JX094500 | Cronobacter phage CR5 | Myoviridae | 223,989 |
| KX397367 | Erwinia phage vB_EamM_EarlPhillipIV | Myoviridae | 223,935 |
| NC_028950 | Ralstonia phage RSL2 | Myoviridae | 223,932 |
| JX100814 | Caulobacter virus Rogue | Siphoviridae | 223,720 |
| NC_028899 | Ralstonia phage RSF1 | Myoviridae | 222,888 |
| NC_023688 | Aeromonas phage PX29 | Myoviridae | 222,006 |
| KT624200 | Bacillus phage SP-15 | Myoviridae | 221,908 |
| NC_019410 | Caulobacter virus Karma | Siphoviridae | 221,828 |
| MG250483 | Aeromonas phage Ah1 | Myoviridae | 221,116 |
| MH588544 | Caulobacter phage CcrBL10 | Siphoviridae | 220,934 |
| MH791407 | Aeromonas phage AsFcp_4 | unclassified | 220,450 |
| KY555143 | Caulobacter phage Ccr2 | Siphoviridae | 220,299 |
| JQ362498 | Sphingomonas phage PAU | Myoviridae | 219,372 |
| KY555142 | Caulobacter phage Ccr10 | Siphoviridae | 219,348 |
| NC_019411 | Caulobacter virus Swift | Siphoviridae | 219,216 |
| MH791401 | Aeromonas phage AsFcp_2 | unclassified | 219,171 |
| NC_009760 | Bacillus phage 0305phi8-36 | Myoviridae | 218,948 |
| JX100812 | Caulobacter virus Magneto | Siphoviridae | 218,929 |
| KY555144 | Caulobacter phage Ccr5 | Siphoviridae | 218,729 |
| NC_023610 | Erwinia phage PhiEaH1 | Siphoviridae | 218,339 |
| KY555147 | Caulobacter phage Ccr34 | Siphoviridae | 216,240 |
| NC_031927 | Synechococcus phage S-CAM7 | Myoviridae | 216,121 |
| KY555146 | Caulobacter phage Ccr32 | Siphoviridae | 215,799 |
| NC_019405 | Caulobacter virus phiCbK | Siphoviridae | 215,710 |
| KU686213 | Synechococcus phage S-CAM7 | Myoviridae | 214,214 |
| AJ697969 | Pseudomonas phage EL | Myoviridae | 211,215 |
| NC_020851 | Synechococcus phage S-SKS1 | Siphoviridae | 208,007 |
| KR052482 | Sinorhizobium phage phiN3 | Myoviridae | 206,713 |
| JX163858 | Caulobacter virus phiCbK | Siphoviridae | 205,504 |
| MF351863 | Synechococcus phage Bellamy | Myoviridae | 204,930 |

# Supplementary Figure 1: MIJ3 titers in each fraction after AKTA purification


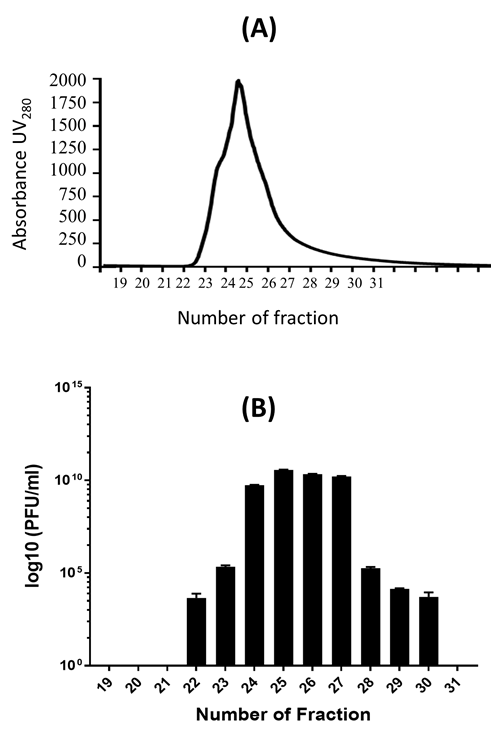


Supplementary Figure 1: Enumeration of phage MIJ3 after AKTA purification. **(A)** A graph represents the number of purified phage fractions. The graph was adjusted from the figure (11A). **(B)** Titers of purified phage MIJ3 in each fraction under the peak.

# Supplementary Table 2: A summary of the host range of phage MIJ3 against lab strains and clinical isolates of *P. aeruginosa.* Zones of lysis scores indications are (1) for complete lysis, (2) for turbid and weak lysis, and (3) for no lysis.

| Strain | Zone of Lysis Score | Single Plaque | Strain | Zone of Lysis Score | Single Plaque |
| --- | --- | --- | --- | --- | --- |
| PAO1 | 1 | Yes | **LES 29** | 3 | No |
| PA14 | 2 | Yes | **LES 30** | 3 | No |
| LES 1 | 3 | No | **LES 31** | 1 | Yes |
| LES 2 | 3 | No | **LES 32** | 1 | No |
| LES 3 | 2 | No | **LES 33** | 2 | No |
| LES 4 | 3 | No | **LES 34** | 2 | No |
| LES 5 | 1 | Yes | **LES 35** | 2 | Yes |
| LES 6 | 3 | No | **LES 36** | 3 | No |
| LES 7 | 1 | Yes | **LES 37** | 2 | Yes |
| LES 8 | 1 | Yes | **LES 38** | 3 | No |
| LES 9 | 3 | No | **LES 39** | 2 | Yes |
| LES 10 | 3 | No | **LES 40** | 2 | No |
| LES 11 | 1 | Yes | **LES400** | 1 | Yes |
| LES 12 | 3 | No | **LES431** | 3 | No |
| LES 13 | 3 | No | **LESB58** | 1 | Yes |
| LES 14 | 2 | Yes | **LESB65** | 1 | Yes |
| LES 15 | 2 | Yes | **LRI 1** | 3 | No |
| LES 16 | 3 | No | **LRI 2** | 1 | No |
| LES 17 | 3 | No | **LRI 3** | 1 | Yes |
| LES 18 | 2 | Yes | **LRI 4** | 3 | No |
| LES 19 | 3 | No | **LRI 5** | 3 | No |
| LES 20 | 3 | No | **LRI 6** | 1 | Yes |
| LES 21 | 1 | Yes | **LRI 7** | 3 | No |
| LES 22 | 1 | Yes | **LRI 8** | 1 | Yes |
| LES 23 | 2 | No | **LRI 9** | 1 | No |
| LES 24 | 1 | Yes | **LRI 10** | 1 | Yes |
| LES 25 | 3 | No | **LRI 11** | 1 | No |
| LES 26 | 2 | No | **LRI 12** | 3 | No |
| LES 27 | 1 | Yes | **COPD** | 1 | Yes |
| LES 28 | 3 | No |  |  |  |

# Supplementary Table 3: A list of the open reading frames (ORFs) in the genome of phage MIJ3 encoding proteins with the identifiable sequence similarity to other proteins. All ORFs encoding hypothetical proteins (303 ORFs) were removed from the table.

| locus_tag | Strand | Start | End | Length (aa) | Annotation |
| --- | --- | --- | --- | --- | --- |
| MIJ3_03 | - | 1172 | 1489 | 317 | Phage tail sheath completion protein |
| MIJ3_04 | - | 1599 | 2969 | 1370 | Tail sheath stabilization protein |
| MIJ3_14 | - | 11832 | 12347 | 515 | Lysozyme RrrD |
| MIJ3_15 | + | 12469 | 12900 | 431 | GatB/YqeY domain protein |
| MIJ3_17 | + | 13114 | 13584 | 470 | Dihydrofolate reductase |
| MIJ3_18 | + | 13581 | 13826 | 245 | putative permease |
| MIJ3_22 | + | 15294 | 15545 | 251 | Glutaredoxin 1 |
| MIJ3_27 | + | 17216 | 19399 | 2183 | RIIA phage protein |
| MIJ3_28 | + | 19392 | 20135 | 743 | RIIB phage protein |
| MIJ3_29 | + | 20219 | 22240 | 2021 | Oligopeptidase A |
| MIJ3_34 | + | 24364 | 25116 | 752 | Phage neck protein |
| MIJ3_35 | + | 25121 | 25762 | 641 | AAA domain protein |
| MIJ3_36 | + | 25880 | 28519 | 2639 | Tail sheath monomer |
| MIJ3_41 | + | 32939 | 33367 | 428 | Head completion protein |
| MIJ3_45 | + | 36057 | 37652 | 1595 | Phage terminase, large subunit |
| MIJ3_51 | + | 43721 | 44992 | 1271 | Protein RecA |
| MIJ3_52 | + | 44985 | 46820 | 1835 | NH homing endonuclease III |
| MIJ3_56 | + | 47794 | 49245 | 1451 | DNA repair helicase RadD |
| MIJ3_64 | + | 53148 | 54176 | 1028 | DNA primase |
| MIJ3_65 | + | 54178 | 55617 | 1439 | DNA repair protein RadA |
| MIJ3_84 | + | 67862 | 70147 | 2285 | Ribonucleoside-diphosphate reductase 1 subunit alpha |
| MIJ3_85 | + | 70201 | 71340 | 1139 | Ribonucleoside-diphosphate reductase 1 subunit beta |
| MIJ3_89 | + | 72918 | 73679 | 761 | Bis(5'-nucleosyl)-tetraphosphatase PrpE |
| MIJ3_92 | + | 74357 | 75253 | 896 | Thymidylate synthase thyA |
| MIJ3_94 | + | 75713 | 76402 | 689 | GTP cyclohydrolase 1 folE |
| MIJ3_95 | + | 76470 | 76988 | 518 | 6-carboxy-5,6,7,8-tetrahydropterin synthase |
| MIJ3_96 | + | 77006 | 78232 | 1226 | ATP-dependent Clp protease ATP-binding subunit ClpX |
| MIJ3_97 | + | 78219 | 78425 | 206 | 30S ribosomal protein S21 |
| MIJ3_99 | + | 78765 | 79235 | 470 | 6-carboxy-5,6,7,8-tetrahydropterin synthase |
| MIJ3_100 | + | 79319 | 79750 | 431 | Small heat shock protein IbpB |
| MIJ3_101 | + | 79866 | 80168 | 302 | ATP-dependent Clp protease adapter protein ClpS |
| MIJ3_102 | + | 80243 | 80791 | 548 | Superoxide dismutase [Mn/Fe] |
| MIJ3_107 | + | 82424 | 84154 | 1730 | ATP-dependent zinc metalloprotease FtsH |
| MIJ3_108 | + | 84182 | 85552 | 1370 | DNA ligase |
| MIJ3_111 | + | 86349 | 87122 | 773 | Exodeoxyribonuclease 10 |
| MIJ3_113 | + | 87613 | 88284 | 671 | ATP-dependent Clp protease proteolytic subunit |
| MIJ3_119 | + | 90482 | 90961 | 479 | tRNA-specific adenosine deaminase |
| MIJ3_130 | + | 94782 | 95291 | 509 | DNA polymerase III alpha subunit |
| MIJ3_132 | + | 95647 | 95973 | 326 | chaperonin |
| MIJ3_133 | + | 95987 | 96916 | 929 | DNA polymerase III subunit |
| MIJ3_136 | - | 97949 | 98722 | 773 | PhoH-like protein |
| MIJ3_139 | + | 100190 | 100879 | 689 | Chaperone protein |
| MIJ3_140 | + | 100889 | 103108 | 2219 | ATP-dependent Clp protease ATP-binding subunit ClpA |
| MIJ3_142 | + | 103675 | 110385 | 6710 | Minor tail protein |
| MIJ3_144 | + | 110889 | 113912 | 3023 | Tail protein |
| MIJ3_149 | + | 120021 | 120593 | 572 | Cytitidyltransferase |
| MIJ3_150 | + | 120596 | 121543 | 947 | N-acetylneuraminate epimerase |
| MIJ3_152 | + | 123537 | 123812 | 275 | Double-stranded DNA binding protein |
| MIJ3_153 | + | 123866 | 126277 | 2411 | DNA polymerase II PolB |
| MIJ3_154 | + | 126270 | 127952 | 1682 | HNH homing endonuclease III |
| MIJ3_155 | + | 127953 | 129092 | 1139 | DNA-polymerase II PolB |
| MIJ3_163 | + | 134207 | 134641 | 434 | Xanthine phosphoribosyltransferase |
| MIJ3_164 | + | 134712 | 135584 | 872 | 7-carboxy-7-deazaguanine synthase |
| MIJ3_167 | + | 136616 | 137602 | 986 | DNA polymerase I |
| MIJ3_177 | + | 141349 | 142464 | 1115 | AAA ATPase |
| MIJ3_181 | + | 143798 | 145294 | 1496 | HNH homing endonuclease III |
| MIJ3_182 | + | 145350 | 146534 | 1184 | DNA repair ATPase |
| MIJ3_187 | - | 148902 | 149504 | 602 | HNH endonuclease |
| MIJ3_207 | + | 161442 | 162008 | 566 | Spore cortex-lytic enzyme |
| MIJ3_217 | + | 166203 | 167630 | 1427 | AAA+ ATPase and BCS1 domain protein |
| MIJ3_229 | + | 174830 | 174906 | 76 | tRNA-Met |
| MIJ3_230 | + | 174929 | 175013 | 84 | tRNA-Leu |
| MIJ3_231 | + | 175064 | 175139 | 75 | tRNA-Asn |
| MIJ3_232 | + | 175166 | 175241 | 75 | tRNA-Thr |
| MIJ3_233 | + | 175311 | 175387 | 76 | tRNA-Met |
| MIJ3_234 | + | 175438 | 175522 | 84 | tRNA-Leu |
| MIJ3_236 | + | 176469 | 176545 | 76 | tRNA-Arg |
| MIJ3_239 | + | 177441 | 177517 | 76 | tRNA-Met |
| MIJ3_240 | + | 177527 | 177603 | 76 | tRNA-Pro |
| MIJ3_241 | + | 177613 | 177686 | 73 | tRNA-Gly |
| MIJ3_244 | + | 178865 | 178954 | 89 | tRNA-Ser |
| MIJ3_245 | + | 178960 | 179049 | 89 | tRNA-Ser |
| MIJ3_255 | + | 182308 | 182538 | 230 | RNA polymerase-binding transcription factor DksA |
| MIJ3_263 | + | 184632 | 185243 | 611 | tRNA(Ile)-lysidine synthase TilS |
| MIJ3_386 | - | 242202 | 243947 | 1745 | Tail fibre protein |
| MIJ3_390 | - | 256288 | 260247 | 3959 | Baseplate wedge |
| MIJ3_394 | - | 261370 | 262803 | 1433 | baseplate hub subunit and tail lysozyme |
| MIJ3_398 | + | 265864 | 266706 | 842 | Sigma factor for late transcription |
| MIJ3_399 | + | 266718 | 267731 | 1013 | Calcineurin-like phosphoesterase superfamily domain protein |
| MIJ3_400 | + | 267731 | 269485 | 1754 | Recombination endonuclease subunit |
| MIJ3_404 | - | 272938 | 273792 | 854 | Baseplate hub subunit |
| MIJ3_406 | + | 276288 | 278003 | 1715 | Portal vertex protein |
| MIJ3_409 | + | 279161 | 279793 | 632 | Phage prohead core protein precursor |
| MIJ3_410 | + | 279806 | 280912 | 1106 | Scaffolding protein |
| MIJ3_411 | + | 280932 | 282119 | 1187 | Major capsid protein |
| MIJ3_412 | + | 282238 | 283815 | 1577 | HNH homing endonuclease III |
| MIJ3_415 | + | 285041 | 285346 | 305 | Carbohydrate binding domain protein |
